# What will and won’t happen when abortion is banned

**DOI:** 10.1093/jlb/lsac011

**Published:** 2022-04-28

**Authors:** Michelle Oberman

**Affiliations:** Santa Clara University School of Law, Santa Clara, CA, USA

**Keywords:** abortion, reproductive justice

## Abstract

For the past 50 years, abortion opponents have fought for the power to ban abortion without little attention to how things might change when they won. The battle to make abortion illegal has been predicated on three nebulous assumptions about how abortion bans work. First, supporters believe banning abortion will deter it. Second, they hope bans will send a message about abortion—specifically, that abortion is immoral. And third, they expect bans to be competently implemented and enforced. Drawing on empirical work from within and outside of the U.S., this Article offers an evidence-based assessment of each of these assumptions. Part One examines the question of deterrence by exploring findings from countries with relatively high and relatively low abortion rates. After explaining why restrictive abortion laws alone do not reduce aggregate abortion rates, I consider the matter of individual deterrence. By identifying those most likely to be deterred by U.S. abortion bans, I illustrate how abortion bans intersect with structural inequalities to disproportionately impact poor women of color and their children. Part Two tests the idea that abortion bans send a message. I consider the bans’ meaning in context with U.S. laws and policies affecting families, exposing the difference between laws discouraging abortion, and those encouraging childbirth. Then, drawing from literature on the expressive function of the law, I assess the limits on the message-sending capacity of abortion bans in a society divided over abortion and over its commitment to children living in poverty. Part Three turns to the expectation that abortion bans will be competently enforced, noting the legitimacy struggles arising from law enforcement patterns, along with the administrative challenges inherent in overseeing the various exceptions to abortion bans. This article concludes by considering why the consequences and limitations of abortion bans should matter to supporters and opponents, alike.

##  

For the past 50 years, abortion opponents have fought for the power to ban abortion without paying much mind to the details of how things might change when they won. The battle to make abortion illegal has been waged over a surprisingly nebulous assumption that banning abortion would, in itself, lead to meaningful changes in the practice of abortion in America. It has been a policy based on hopes and prayers, rather than on actual evidence about how restrictive abortion laws work in practice.

When the law on the ‘books’ changes in the United States, what might the law on the ground look like? Drawing on empirical work from within and outside of the USA, this article offers an evidence-based answer to the question of what will and would not happen where abortion is banned.

In the spirit of full disclosure, like almost everyone who engages with the abortion war, I have a bias: I am an unambivalent supporter of abortion rights. Nonetheless, I strive in this article to maintain a tone that I hope will permit readers who disagree with me to hear my message. I do so because those on all sides of our abortion war should care about it. For 50 years, the USA abortion war has been fought almost exclusively around the issue of legalization.^1^ Yet all evidence suggests the changes likely to be wrought by banning abortion should leave even ardent supporters of abortion bans not just disappointed, but profoundly disturbed by their downstream consequences.

The question of how abortion bans work in practice is a live one among abortion-rights advocates, with many, (including myself), working to identify what happens, and to whom, so as to permit advocates and policy makers to mitigate their harsh impact on the vulnerable.^2^ By contrast, anti-abortion Americans who have spent decades working to enact such laws have paid relatively little attention to how things actually change when abortion is illegal. Instead, they have rested their support for outlawing abortion on three general assumptions about how abortion bans will work. First, they believe banning abortion will deter it.^3^ Second, they hope the bans will send a message about abortion—specifically, that abortion is immoral.^4^ And third, they expect the laws will be competently implemented and enforced.^5^

This article interrogates each of these expectations. Part One begins its consideration of the question of deterrence by exploring research from countries with relatively high and relatively low abortion rates. This comparison offers powerful evidence that restrictive abortion laws alone do not reduce abortion rates. But while outlawing abortion is unlikely to cause an aggregate decline in abortion rates, bans will cause some to carry to term pregnancies they might otherwise have aborted.^6^ This section concludes with an examination of how abortion bans intersect with structural inequalities to disproportionately impact poor women of color and their children–already the most vulnerable and marginalized Americans.

Part Two tests the assumption that outlawing abortion will send a message that abortion is morally wrong, thereby helping to foster a culture that rejects abortion as an option. This section considers the messages sent by US abortion bans by placing them in context with our laws and policies that inform when and whether people seek abortions. Then, drawing from literature on the expressive function of the law, this section explores the practical and symbolic limits on the message-sending capacity of abortion bans in a society divided over abortion and over its commitments to children living in poverty.

Part Three moves to the expectation that abortion bans will be competently enforced. Here, I examine the legitimacy struggles arising from law enforcement patterns, along with the administrative challenges inherent in overseeing the various exceptions to abortion bans.

This article concludes with a consideration of why the consequences and limitations of abortion bans should matter to supporters and opponents, alike.

## I. THE HOPE THAT ABORTION BANS WILL DETER ABORTION

Abortion opponents anticipate that banning abortion will deter it. That said, they are not overly sanguine about this hope; anti-abortion advocates acknowledge that abortions will continue to take place, even if illegal.^7^ But their support for abortion bans rests firmly on the expectation that there will be fewer of them, once abortion is a crime.^8^ In this section, I explore the question of deterrence, first considering the population-wide impact of bans on abortion rates, and then describing the Americans most likely to be deterred by abortion bans.

### I.A. Deterrence, in the Aggregate

To think about whether abortion can be deterred by outlawing it, we must begin by reflecting on what leads people to have abortions. Abortion demand is driven by a host of factors—health status, relationship status, job status—but the most commonly cited concern is lack of money.^9^ Half of all US abortions go to the 13 per cent of Americans living below the poverty line–which in 2022, means living on less than $13,590.^10^ Those living in poverty or near poverty make up a full 76 per cent of abortions every year.^11^ These are people whose abortion decisions are motivated, at least in part, because they cannot afford the costs of child rearing.^12^

Rather than focusing on reducing abortion demand by offsetting the costs of having children, abortion bans aim to deter abortion solely by reducing access to legal abortion. Even a cursory glance at worldwide abortion rates suggests that strategy might not work. Abortion rates vary dramatically by region. In Latin America, (home to countries with the world’s strictest abortion bans), we find some of the highest abortion rates in the world: 32 abortions for every 1000 women.^13^ At the other end of the spectrum, Western Europe, (with relatively liberal abortion laws), has the world’s lowest abortion rates: only 12 abortions per 1000 women.^14^

The variation in abortion rates is best understood as an artifact of variation in rates of unintended pregnancy.^15^ The single biggest predictor of abortion rates is not the legal status of abortion, but rather, the percentage of pregnancies that occur among those who were not looking to have a baby. In 2014, the most recent year for which data is available, 44 per cent of pregnancies globally were unintended. 53 per cent of those unintended pregnancies ended in abortion.^16^ Rates of both unintended pregnancy and abortion vary by a country’s wealth status. In the world’s wealthier nations, over the past quarter century, rates of unintended pregnancies dropped by 30 per cent, triggering a decline in abortion rates from 46 abortions per 1000 women of reproductive age to an average of 27.^17^ By contrast, over the same time frame in the developing world, unintended pregnancy rates fell by only 16 per cent, while abortion rates remained static.^18^

Like other wealthy countries, U.S. abortion rates have dropped significantly in recent decades.^19^ The decline is evident across almost every demographic in the country—younger, older, Northern, Southern. With one exception: abortion rates have remained constant among the poorest Americans.^20^ This finding underscores the significance of unintended pregnancy in driving abortion rates: nearly half of all pregnancies in the United States are unintended—a higher rate than in many other developed countries.^21^ These rates vary dramatically by class: a poor woman in the USA is more than five times as likely as an affluent woman to have an unintended pregnancy.^22^

The single most effective way to help people avoid unwanted pregnancies, thereby deterring abortion, is by increasing contraception rates. When the Affordable Care Act mandated insurance coverage for contraception, the unintended pregnancy rate dropped from 44.7 to 37.9 per cent.^23^ And yet, the anti-abortion movement has opted to oppose efforts to increase access to contraception.^24^ Indeed, abortion opponents vigorously fought the Affordable Care Act’s birth control mandate, which the Supreme Court ultimately struck down in 2014.^25^

If the goal of banning abortions is to deter them, a strategy that fails to focus on reducing unintended pregnancy seems limited, at best. But even if one accepts that abortion opponents are too ambivalent about promoting contraception to center the goal of deterring abortion by reducing unwanted pregnancy, the plan to deter abortion by banning it is flawed for a second reason. Specifically, owing to the ready availability of abortion medicines, abortion bans cannot effectively restrict access to a safe, effective, and affordable means to end a pregnancy.

The widespread availability of abortion medicines has completely transformed the world of illegal abortion. Unlike the pre-Roe era, medication abortion solves the problem of finding a doctor to perform an illegal abortion, while simultaneously reducing the health risks.^26^ The most common and widely available abortion medicine is misoprostol.^27^ Although less effective than the FDA-approved combination of mifepristone and misoprostol typically used in medical abortions in the USA, misoprostol alone causes an abortion in approximately 90 per cent of cases.^28^ Efforts to restrict access to misoprostol are complicated for two reasons. It is both cheap and easy to manufacture, costing only pennies to make, and it also is an important life-saving medicine.^29^ Indeed, the World Health Organization lists misoprostol as an ‘essential medicine,’ owing in part to its vital role in reducing deaths from postpartum hemorrhages, miscarriages, and illegal abortions.^30^

There is a robust international market in misoprostol across the world today—particularly in countries where abortion is strictly banned.^31^ Even in Central America, which boasts the world’s strictest abortion bans, one in three pregnancies ends in abortion, largely induced by medicines purchased online or on the street.^32^ Americans familiar with the black market in opioids should have little trouble imagining how a market in abortion medicines will proliferate, where abortion is banned. As is all too evident from the scope of the opiate problem, it is unrealistic to think the government can prosecute away the expanding market in abortion medicines.^33^

Outlawing abortion may lead to a short-term decline in US abortion rates, while people adjust to new market conditions.^34^ But as we learn from the experiences of countries throughout the world, this decline is unlikely to be sustained. If anything, given the availability of reliable online information and buying options, it should take far less time for people to adapt to accessing illegal abortion than was true for alcohol access after Prohibition.^35^

### I.B. Deterrence, in the Specific

Even if abortion bans are unlikely to cause an aggregate decline in abortion rates–at least not independently of other trends^36^–we can predict that they will cause some to carry to term pregnancies they might otherwise have aborted.^37^ In fact, we have a surprisingly clear picture of those who the bans are most likely to deter: they will be disproportionately young, poor, Black, and brown women. Abortion bans come as one in a long list of factors that circumscribe the reproductive lives and life options of these Americans.^38^ They are more likely to experience unintended pregnancy, and where abortion is outlawed, they are more likely to struggle with accessing abortion, whether by traveling to a legal jurisdiction, or by identifying reliable information about how to safely end an unwanted pregnancy with abortion medications.^39^

Those who support abortion bans on deterrence grounds have yet to fully grapple with what happens to those for whom abortion, legal or otherwise, is out of reach. The standard response is to promote the solution of placing newborn babies for adoption. Justice Amy Coney Barrett nodded to this viewpoint at oral argument in the Dobbs case, correcting the assertion that abortion bans amount to ‘forced motherhood’ by noting that safe haven laws permit them to surrender their newborns without legal consequences.^40^ Adoption proponents point to the ways in which open adoptions have become the norm, hopeful that the prospect of staying involved in their baby’s life will encourage more people to place them for adoption.^41^ Banning abortion, as they see it, can be a ‘win-win-win’ situation, in which the baby survives, the mother gets to go on with her life, and a married couple or family gets to raise the child.^42^

Yet all available evidence suggests that banning abortion is unlikely to transform adoption from an outlier into a commonplace response to unwanted pregnancy. Even in the years prior to Roe, when the stigma of unwed motherhood led some facing pregnancy to place their babies, only 9 per cent of women chose adoption.^43^ Much of that rate was driven by white women, because the two-parent family norm was less entrenched among Black and brown Americans. Today, the stigma is gone: 40 per cent of all children are born out of wedlock.^44^ When faced with an unintended pregnancy, fewer than 5 per cent of people seriously consider adoption, and of those, fewer than 2 per cent ultimately place their children with adoptive families.^45^

The best indication of what is likely to happen to those unable to access abortion is found in the Turnaway study, a 10-year longitudinal investigation of the impact of being denied an abortion.^46^ That study followed hundreds of women who sought abortions, but were turned away because they were beyond the clinic’s gestational limits.^47^ Fully 91 per cent of them opted to raise their child.^48^

The Turnaway study also tells us about the consequent intensification of poverty for these families:

[C]hildren of women who are denied an abortion had greater odds (72 vs 55%) of living in poverty compared to children of women who received a wanted abortion. Similarly, existing children were more likely (87 vs 70%) to live in a household in which their mother is not able to afford necessary living expenses such as food, housing, and transportation compared to children of women who received a wanted abortion.^49^

As we look to understand what happens when an abortion ban ‘works’ by deterring abortion, the only question is how broad a lens to use. More than one in three single-mother families lived in poverty in 2016.^50^ Poverty is not color-blind. Instead, far more women of color live in poverty than do their white counterparts: close to 25 per cent of all American Indian or Alaskan native and 20 per cent of all Black and Hispanic women live in poverty, compared to only 9 per cent of their white counterparts.^51^

So severe are the downstream consequences for children born into poverty that the American Academy of Pediatrics issued a statement decrying the short and long-term consequences of the ‘medicalization of poverty’:

Children who experience poverty, particularly during early life or for an extended period, are at risk of a host of adverse health and developmental outcomes through their life course. Poverty has a profound effect on specific circumstances, such as birth weight, infant mortality, language development, chronic illness, environmental exposure, nutrition, and injury. Child poverty also influences genomic function and brain development…. Children living in poverty are at increased risk of difficulties with self-regulation and executive function, such as inattention, impulsivity, defiance, and poor peer relationships. Poverty can make parenting difficult, especially in the context of concerns about inadequate food, energy, transportation, and housing. Child poverty is associated with lifelong hardship. Poor developmental and psychosocial outcomes are accompanied by a significant financial burden, not just for the children and families who experience them but also for the rest of society….^52^

We should read these statistics as a forecast. To the extent abortion bans deter abortion, we will likely see a disproportionate increase in the number of poor families of color experiencing the devastating consequences of living in poverty. Abortion bans work by leveraging existing inequalities.^53^

Abortion opponents are of two minds about how to respond to the poor predicted outcomes for those who opt to raise children after being unable to access abortion. Small numbers of advocates–largely drawn from the volunteer ranks of pregnancy support or ‘crisis pregnancy’ centers–advocate helping women who are in desperate straits by offering housing, counseling, job training, and other support.^54^ But the dominant voice of the anti-abortion movement–those advocates engaged in political activism and law reform, rather than direct service–focuses not on supporting poor mothers, but instead, on promoting adoption.^55^

The suggestion that adoption is the optimal solution to a poorly timed pregnancy is as convenient as it is naïve. It allows abortion opponents to avoid a reckoning with consequences of having made the Republican party their political home.^56^ The GOP’s historical and ongoing objection to family-friendly government policies^57^ will make it hard, in the years to come, for abortion opponents to gain much traction for laws aimed at blunting the crushing impact of poverty on those whom the bans deter from having abortions.

## II. THE HOPE THAT ABORTION BANS WILL SEND A MESSAGE

Those who support abortion bans do not rest their support solely on the expectation that such laws will deter abortion. Instead, abortion opponents often invoke the belief that changing the law will send a message, thereby promoting culture change.^58^ This section first considers the nature of that message, and then turns to whether it will be received.

It is helpful, when considering the message sent by outlawing abortion, to note the difference between an anti-abortion message (one that condemns abortion) and a pro-natal message (one that urges people to have babies). This distinction is easiest to observe when contrasting U.S. laws with those of countries that actively encourage childbearing.

Consider the case of Israel, which makes abortion a crime unless the person can prove to an official ‘pregnancy termination committee’ that they qualify for one of the statute’s exceptions.^59^ This law would send a message that, outside of exceptional circumstances, abortion is wrong.^60^ But it also exists alongside a host of laws and policies that encourage people to have children.^61^ In Israel, there is guaranteed paid maternity leave—you can leave your job for 26 weeks, still get paid, and your employer cannot fire you.^62^ Parents enjoy access to local neighborhood, government-subsidized day care.^63^ In addition to tax deductions, the Israeli government pays everyone—rich and poor alike—a small monthly allowance for each child under eighteen.^64^

In addition to the ways in which these policies help offset some of the most immediate costs associated with having a child, they send a message about how the government feels not just about abortion but also about babies. Israel’s laws send a message that the government wants people to have babies.

By contrast, US laws reflect little interest in encouraging people to have babies, particularly ones they cannot afford to raise. There is no paid parental leave, and no job security at all beyond the first 12 weeks of unpaid leave.^65^ There is no child allowance. The Covid-related child income tax break, which reduced child poverty by 30 per cent, was permitted to lapse after a single year.^66^ The goal of providing universal access to quality day care and preschool remains a pipedream.^67^ The federal assistance program, Temporary Assistance to Needy Families, is so under-funded that no state’s subsidy amounts to more than 60 per cent of the federal poverty line, with the result that even in states with relatively generous monthly allocations, families cannot afford modest rent.^68^

Those who believe abortion bans promote a culture of life might do well to recognize that any message sent by an abortion ban is necessarily entwined with the messages sent by government laws and policies that set the price of having a child. The message of an abortion ban on its own says little about embracing life, and instead merely suggests that abortion is wrong.^69^

As to whether that message will be received, the answer is complicated. For all that, it is common to suggest the law can send messages, there is surprisingly little evidence for how it might do so. Professor Richard McAdams, one of the leading authorities on the ‘expressive function’ of the law, posits that an expressive law reveals the lawmakers’ beliefs, which in turn causes individuals to update their beliefs and ultimately to change their behaviors, usually in the direction of compliance.^70^ He points to the example of indoor smoking bans by way of illustration. In the face of mounting evidence on the harmfulness of secondhand smoke, lawmakers enacted indoor smoking bans that served, in part, to send the message that tobacco was dangerous. In turn, these bans helped shift the culture away from smoking.^71^

According to McAdams, smoking bans succeeded because lawmakers had a clear, credible message. But government credibility is not automatic; rather, it is earned. To send a message, government actors must offer some reason why the public should trust their conclusions. McAdams suggests the government earned credibility by persuading the public they were acting on data showing that the hazards of secondary smoke inhalation required nothing less.^72^

Unlike smoking bans, abortion bans address themselves to a question of morality—one that cannot be settled by aggregated data or special expertise. A government hoping to persuade the public that abortion is immoral will struggle simply because it lacks the expertise needed to settle the question.^73^

The challenge of sending a message by banning abortion is intensified by the impact of the ongoing battle over abortion’s legality. When it comes to the law’s ability to send a message, Professor McAdams notes, background noise can be fatal:

Individuals are constantly being bombarded by information from sources other than the law: the print media, Internet, social acquaintances, etc. For expression to change beliefs, there must be some factor that makes the legal signal strong enough to stand out against this background. ^74^

To send a message, abortion bans must compete for air time with a world of counter-messages. After all, the fight over abortion does not end with abortion bans. Together with the likelihood that abortion remains commonplace, even where banned, and remains legal in almost half the country, the message-sending capacity of abortion bans is more akin to that of marijuana bans than to indoor smoking bans.^75^

At the end of the day, perhaps the most that can be said for the message-sending capacity of abortion bans is that, where popularly embraced by an anti-abortion electorate, the bans might contribute to a broader cultural message that abortion is wrong. As Katrina Kimport forcefully demonstrates in her book, *No Real Choice*, the ‘abortion as killing’ narrative can combine with structural constraints like legal barriers and cost to render abortion ‘unchoosable.’^76^

## III. THE HOPE THAT ABORTION BANS WILL BE COMPETENTLY IMPLEMENTED AND ENFORCED

The final set of expectations harbored by those who support outlawing abortion involves tacit baseline assumptions about how the law will work, in practice. Specifically, supporters assume that abortion bans will be competently implemented and enforced—that the laws will have integrity. Competent implementation and enforcement are not abstract ideals, but rather, are necessary preconditions for a law to be considered a legitimate exercise of state authority. The failure to competently implement an abortion ban will undercut its legitimacy, thereby undermining both the law’s capacity to deter abortion and also its ability to send a message.

To understand the practical considerations relevant to enforcing abortion bans, begin by noting what is required in order to implement them. The standard form of US abortion bans includes a general prohibition, accompanied by a small number of exceptions.^77^ This structure gives rise to two implementation and enforcement questions, both of which will determine whether the laws are ultimately seen as legitimate exercises of government authority. When and how will prosecutors endeavor to enforce the bans, and by what mechanisms will states evaluate cases involving exceptions to the bans?

Let us examine each of these in turn.

### III.A. Enforcing Abortion Bans

Supporters of abortion bans have given relatively little thought to the question of how abortion laws will be enforced. In late 2021, movement leader Marjorie Dannenfelser, President of the Susan B. Anthony List (a nonprofit that supports pro-life politicians) explained how she views the question of enforcement:

[M]y view, and the view of the entire movement—without any exception that I’m aware of—is that the doctor, the one who has been planning to break the law, is the guilty party. The law is enforced against that person, not the woman.^78^

But illegal abortion today need not involve a doctor or any third party besides an overseas pharmacy, outside the easy reach of US laws.^79^ Given abortion medicines, the reality is that there are no doctors to prosecute.

When abortion becomes a crime, the question of who is the criminal will require an answer. And rather than being answered by movement leaders, the decision will rest in the hands of locally-elected prosecutors. No county can afford to prosecute every crime–far from it–so local District Attorneys set priorities when enforcing the law.^80^ Their choices may be informed by many factors: staff resources, strength of evidence, heinousness of crime, perception of public will, or say, pro-choice or anti-abortion sentiment. As Judge Stephanos Bibas notes, there is no check on ‘idiosyncratic prosecutorial discretion.’^81^

A quick review of abortion prosecutions both historically and today helps us understand what idiosyncratic abortion prosecutions might look like. Historian Leslie Regan’s work documents the episodic nature of abortion prosecutions in the years prior to *Roe*, showing how they tended to be sporadic—an occasional crackdown, motivated by a zealous prosecutor, rather than a comprehensive effort at enforcement.^82^

A similar pattern is seen today in places where abortion is outlawed. For example, consider El Salvador, which bans abortion without exception. In the 10 years from 2000–2010, there were 129 prosecutions.^83^ This number suggests enforcement is relatively rare—just over 10 prosecutions per year—when, by the government’s own estimates, the country sees tens of thousands of abortions every year.^84^ But there is a pattern to the prosecutions. Those charged with abortion crimes are drawn from the most vulnerable, marginalized sectors of society.^85^ Almost half were illiterate; only a quarter had attended high school.^86^

In the U.S. we already see a version of this pattern: abortion-related prosecutions are brought by zealous prosecutors^87^, and they disproportionately target Black and brown women.^88^ The work of National Advocates for Pregnant Women helps us to understand the scope of abortion-related prosecutions in the years since Roe legalized abortion. They have tracked 1600 USA such cases since 1973.^89^ These cases involve a range of allegations, linked by the common thread of alleged harm to a pregnancy.^90^ The prosecutions overwhelmingly target poor people, and in particular, poor Black pregnant women. Of 413 cases arising from 1973 to 2005, 71 per cent involved low income women, of whom 59 per cent were women of color, with 52 per cent identifying as Black.^91^

These patterns in abortion-related prosecutions tell us two important things. First, we can expect abortion bans to be enforced against those who end their own pregnancies.^92^ And second, abortion prosecutions are likely to target the most marginalized, vulnerable members of society—those whom prosecutors view, or at least believe others will be willing to view, not as victims but rather, as villains.^93^

Supporters of abortion should stop insisting that bans won’t be enforced against women, and should start figuring out what to do about the fact that, when abortion bans are enforced, the defendants will likely be Black and brown. It is, of course, unfair to make one subset of the population pay the price for acts that go unpunished when committed by others. Furthermore, as we learn from racial disparities in drug law enforcement, such patterns undermine the legitimacy of the law, and have downstream corrosive effects both on the people disproportionately targeted by the law and on society as a whole.^94^

### III.B. The Return of Conditional Abortion Access

Setting aside the question of prosecution, abortion laws also must be fully implemented in the regulatory sense of the word. A law that limits abortion access to patients with qualifying conditions presupposes an adjudicatory mechanism for determining eligibility. And barring a dramatic evisceration of the right to life for those who are pregnant, every state will have to make at least one exception to their abortion bans, for life-threatening pregnancies.^95^

How will a patient establish their right to an abortion when they are experiencing a life-threatening pregnancy?

There are a variety of models by which states might screen such claims, ranging from relatively formal proceedings, such as those seen in cases involving termination of government benefits, to loosely structured processes like school disciplinary hearings.^96^ Indeed, we already have a model for abortion-related adjudications in the judicial bypass system, by which minors can seek permission to end a pregnancy without parental involvement.^97^

Each model is fraught, when it comes to screening for abortion eligibility. Formal judicial hearings pose challenges in terms of accuracy (there is surprisingly little agreement on what constitutes a life-threatening pregnancy)^98^ and efficiency (given the urgent, technical nature of the inquiry).^99^ A judge could not conceivably rule on such petitions without expert testimony, which raises numerous questions about process and evidence.

Prior to *Roe*, rather than ask judges to decide these cases, states delegated the determination to doctors, essentially leaving the medical profession to devise its own ways of complying with the law.^100^ For reasons ranging from lack of consensus about qualifying conditions,^101^ to concern over the legal implications of their decisions (which might trigger prosecution on the one hand, or a wrongful death suit if the pregnant patient dies, on the other),^102^ doctors eschewed this responsibility. By the mid-20th century, hospitals around the country used so-called ‘therapeutic abortion committees’ to establish eligibility.^103^ These committees were marked by inconsistent outcomes, stemming from a lack of consensus over what constituted a ‘valid’ reason for terminating a pregnancy, whether legally or morally.^104^ Rather than standardizing the application of the law, the committee process facilitated ad hoc decision-making.^105^

As states set about banning abortion, it is urgent that they erect a scientifically sound, impartial process by which to evaluate cases involving potentially life-saving abortions. Given that the vast majority of Americans support abortion in cases of life-threatening pregnancy, we can expect an enormous outcry from all quarters in the case of an incompetent oversight process, let alone a highly publicized death.^106^

Yet the struggle to define what constitutes a life-threatening pregnancy, (or depending upon the law, a qualifying rape or fetal anomaly), is just the start. Which parties’ interests will be represented at these adjudicatory proceedings; however, they are configured? Will the pregnant patient be entitled to a lawyer? ^107^ Will the fetus? If unhappy with the outcome, can either side appeal? Will there be an expedited appeals process? By what criteria will adjudicators be chosen? Will these be adversarial proceedings, with experts from the state and from the pregnant patient’s medical team, or will the patient’s doctor’s testimony suffice? How will the government determine whose interests it represents: those of the patient in peril, or those of the fetus?

These are serious questions, made all the more so because they implicate vital interests and therefore trigger Constitutional due process rights.^108^ Surely, there will be litigation over the answers in the years to come. But what is interesting about these questions is not so much their answers, but instead, the reality that they demand answers now. We are past the time when those who support banning abortion can respond to such questions about how the laws will be implemented with vague references to ‘traditional means of enforcement.’^109^ And the quality of those answers matters because inconsistent, incompetent or otherwise corrupt law enforcement cannot help but undermine the legitimacy of abortion bans.

## IV. CONCLUSION

We have spent half a century reckoning with abortion largely in abstractions, fighting over rights rather than focusing on the people whose lives are affected by those rights. If nothing else, the impact of abortion bans seems likely to put human faces on the abortion war. And if we stay true to the patterns laid out in this essay, those faces will be disproportionately poor, Black and brown women and children.

Abortion bans are not color blind.

It has become common for abortion opponents to invoke allegations of eugenics and racism when talking about abortion rates among Black Americans.^110^ That rhetoric–already contested^111^–will become strained as the country witnesses the actual racist impact of abortion bans: their disparate impact on poor Black families, coupled with the disparate rates of prosecution of Black women for acts that go largely unpunished when committed by whites.^112^

By bringing into focus the struggles facing the most vulnerable among us, abortion bans have the potential to transform the abortion war by forcing a direct engagement with the structural forces driving abortion, poverty, and racism. We are approaching a moment of truth for advocates on all sides of the abortion war.

For advocates of abortion rights, there will be a reckoning with the question of whether being pro-choice simply means supporting the right to abortion, rather than a commitment to working to offset the forces that constrain all reproductive options–including having a child. As Sister Song, a leading voice of the reproductive justice movement puts it, the commitment is to support, ‘the human right to maintain personal bodily autonomy, have children, not have children, and parent the children we have in safe and sustainable communities.’^113^ As the impact of abortion bans brings structural inequality into sharp focus, will pro-choice movement leaders stay focused on legalizing abortion, or will the movement commit to this more robust understanding of reproductive autonomy?

For abortion opponents, the question is whether the term ‘pro-life’ has come to mean anything beyond one’s support for abortion bans. The years ahead likely will pose an existential challenge for people who have supported abortion bans, but who cannot help but be disturbed by the ways in which they fall short of expectations. Perhaps this result will embolden those who care deeply about deterring abortion, and find them laboring to craft policies that might actually help those contemplating abortion to continue their pregnancies.^114^

Certainly, anti-abortion movement leaders are aware of the need to do something proactive in response to the impact of abortion bans on the poor. As Marjorie Dannenfelser, of the Susan B. Anthony Fund, put it: ‘Speaking for the pro-life movement, which is obviously attempting to lead Republicans, we absolutely, without question, have a responsibility to serve the needs of women and children as we pass ambitious laws. There’s no question about it.’ At the same time, Dannenfelser is aware that such policies are unlikely to fly, at least not in states dominated by a Republican party that has long opposed family-friendly government programs.^115^

There’s a quote I keep on my desk these days: “How will we go when we’re faced with this? I don’t think it’s predetermined, and a great human moral drama is being played out in front of us.”^116^ It is from a historian of pandemics, written in the early days of Covid-19. I keep it there because it speaks to me as we navigate the era of abortion bans. There is comfort in the invitation to step back and notice that we are in a time of high moral drama, in which things are in flux. But there is also, within it, a call to action. “How will *we* go when we’re faced with this?”

## Acknowledgements

For helpful suggestions and conversation, I’m grateful to Diana Greene Foster, Julia Hejduk, Carole Joffe, Katrina Kimport, Larry Marshall, and my anonymous reviewers. I was particularly lucky to work with Jenai Howard (SCU Law, 2022), who provided outstanding research assistance. All errors are my own.

## Ethics Approval Statement

Human Subjects Research for this article was approved by Santa Clara University’s IRB, Protocol 17-03-950.

## Funding

Research was funded in part by a Hackworth Grant (Santa Clara University, Markkula Ethics Center).

## Footnotes

1 See Mary Ziegler, After Roe: The Lost History Of The Abortion Debate (2015), for a rich history of the anti-abortion movement in the early years after Roe v. Wade, illustrating among other things the way the anti-abortion movement shifted its focus from efforts to support pregnant women to the narrow issue of criminalization.

2 See generally Michelle Oberman, *How Abortion Laws Do and Don’t Work*, 36 Wis. J. L. Gender & Soc’y 163 (2022); see also Michelle Oberman, Her Body, Our Laws: On The Front Lines Of The Abortion War, From El Salvador To Oklahoma (2018); Diana Greene Foster, The Turnaway Study: Ten Years, A Thousand Women, And The Consequences Of Having Or Being Denied An Abortion (2020) [hereinafter, greene foster, the turnaway study]; Ushma Upadhyay, Alice F. Cartwright, and Daniel Grossman, *Barriers to Abortion Care and Incidence of Attempted Self-Managed Abortion Among Individuals Searching Google for Abortion Care: A National Prospective Study*, 106 Contraception 49 (2021); Lizzie Widdecombe, *What Does an At-Home Abortion Look Like in 2021*, The New Yorker (Nov. 11, 2021), https://www.newyorker.com/news/news-desk/what-does-an-at-home-abortion-look-like-in-2021 (profiling self-managed abortion researcher, Abigail Aiken).

3 For example, see Ross Douthat, *The Case Against Abortion*, N.Y. Times (Nov. 30, 2021), https://www.nytimes.com/2021/11/30/opinion/abortion-dobbs-supreme-court.html (celebrating the impact of Texas’ S.B. 8, credited with causing a 93 per cent drop in the number of abortions taking place in the state).

4 See eg Richard Garnett, *One Untrue Thing*, Nat’l Rev. (Aug. 1, 2007), (https://www.nationalreview.com/2007/08/one-untrue-thing-nro-symposium/ (‘The point of criminalization, after all, is not merely to put people in prison, or deter people from engaging in harmful behavior. It is, instead, to make a statement—a public statement, in the community’s voice—that certain actions, or certain harms caused, are morally blameworthy.’). See also Oberman, *supra* note 2, at 85–86 (quoting an anonymous Oklahoma state senator): The purpose of the law is to stop abortion. To send a moral message. To get the message out via the law, to spark a debate in the population. The government’s responsibility is to give people education. It is up to the government to tell them that abortion is wrong. It’s not an acceptable solution.

5 This assumption is largely a tacit one, inherent in assertions about how abortion bans will be received by the public, and how they are likely to inspire others states to follow suit. See eg Issac Chotiner, *The Pro-Life Movement Plans for a Future Without Roe*, The New Yorker (Dec. 7, 2021), https://www.newyorker.com/news/q-and-a/the-pro-life-movement-plans-for-a-future-without-roe (interviewing Marjorie Dannenfelser, president of the Susan B. Anthony List, on her expectations for passing abortion bans in 30 states).

6 See Diane Greene Foster, *Stop Saying That Making Abortion Illegal Won’t Stop People From Having Them*, Rewire News Group (Oct. 4, 2018) [hereinafter Green Foster, *Stop Saying*], https://rewirenewsgroup.com/article/2018/10/04/stop-saying-that-making-abortion-illegal-doesnt-stop-them/; see generally Greene Foster, The Turnaway Study, *supra* note 2.

7 See eg Stephanie Ranade Krider, *Pro-life Advocates Focused on Legal Battles. They’re Not Enough to End Abortion*, Wash. Post (Oct. 15, 2021), https://www.washingtonpost.com/outlook/pro-life-after-roe/2021/10/15/7e2a059e-2cf8-11ec-985d-3150f7e106b2_story.html (Krider, an abortion opponent, acknowledges that while ‘the end of *Roe* would be a victory and a cause for celebration for those…who oppose abortion, [it] would not end the practice nationwide.’).

8 See Douthat, *supra* note 3.

9 See Biggs, M. Antonia, Heather Gould & Diana Greene Foster, *Understanding why Women Seek Abortions in the US*, 13 BMC Women’s Health 1–13 (2013); see also Sophia Chae et al., *Reasons Why Women Have Induced Abortions: A Synthesis of Findings From 14 Countries*, 96 Contraception 233–41 (2017). (Analyzing data from 14 countries to identify the primary reasons given for seeking abortion, and finding that, although people often listed several reasons, the dominant reason involved socioeconomic concerns).

10 See Annual Update of the HHS Poverty Guideline, 87 Fed. Reg. 3315, 3316 (Jan. 21, 2021).

11 See Rachel K. Jones & Jenna Jerman, *Population Group Abortion Rates and Lifetime Incidence of Abortion: United States, 2008–2014,* 107 Am. J. Publ. Health 1904–909 (2017), https://ajph.aphapublications.org/doi/10.2105/AJPH.2017.304042; see also *Abortion Rates by Income Level*, *Infographic*, Guttmacher Inst. (Oct. 19, 2017), https://www.guttmacher.org/infographic/2017/abortion-rates-income.

12 More accurately, because almost 60% of those having an abortion already have at least one child, they cannot afford the costs of another child. Jerman J, Jones RK & Onda T, *Characteristics of U.S. Abortion Patients in 2014 and Changes Since 2008*, Guttmacher Inst. (2016), https://www.guttmacher.org/report/characteristics-us-abortion-patients-2014.

13 Bearak J et al., *Unintended pregnancy and abortion by income, region, and the legal status of abortion: estimates from a comprehensive model for 1990–2019*, 8 lancet global health 9 (2020).

14
*Id*.

15 An unintended pregnancy is one that occurred when a woman wanted to become pregnant in the future but not at the time she became pregnant (‘wanted later’) or one that occurred when she did not want to become pregnant then or at any time in the future (‘unwanted’). See *Unintended Pregnancy in the United States*, Guttmacher Inst. (Jan. 2017), https://www.guttmacher.org/fact-sheet/unintended-pregnancy-united-states. In recent years, this frame has been problematized by calling into view the reality that, for many people, pregnancies are not so much planned as they are responded to. That is to say, the relevant question is not whether it was intended, but whether it is wanted or unwanted. See Abigail R.A. Aiken, et al., *Rethinking the Pregnancy Planning Paradigm: Unintended Conceptions or Unrepresentative Concepts?*, 48 Perspect Sex Reprod Health 147–151 (2016).

16 See generally *Unintended Pregnancy Rates Declined Globally from 1990 to 2014*, Guttmacher Inst. (Mar. 5, 2018), https://www.guttmacher.org/news-release/2018/unintended-pregnancy-rates-declined-globally-1990-2014.

17
*Id*.

18
*Id*.

19 Between the 1990s and 2020, abortion rates declined by almost 40%. See Sabrina Tavernise, *Why Women Getting Abortions Now Are More Likely to Be Poor*, N.Y Times (July 9, 2019), https://www.nytimes.com/2019/07/09/us/abortion-access-inequality.html. In the years since 2010 alone, rates have declined by almost 20%. Elizabeth Nash & Joerg Dreweke, *The U.S. Abortion Rate Continues to Drop: Once Again, State Abortion Restrictions Are Not the Main Driver*, Guttmacher Inst. (Sept. 18, 2019), https://www.guttmacher.org/gpr/2019/09/us-abortion-rate-continues-drop-once-again-state-abortion-restrictions-are-not-main. Scholars are divided in their explanations for the decline, which vary from increasingly effective contraceptive practices to declines in rates of sexual activity. See Pam Belluck, *America’s Abortion Rate Has Dropped to its Lowest Ever*, N.Y. Times (Sept. 20, 2019), https://www.nytimes.com/2019/09/18/health/abortion-rate-dropped.html; see also Diana Greene Foster, *Dramatic Decreases in US Abortion Rates: Public Health Achievement or Failure?*, 107 Am J. Public Health 1860 (2017); Alia E. Dastagir, *Fewer Women are having abortions. Why?*, USA Today (June 13, 2019), https://www.usatoday.com/story/news/nation/2019/06/13/abortion-law-fewer-women-having-abortions-why/1424236001/; Doug Stanglin, *US Abortion Rate is at its Lowest, but Restrictive Laws aren’t the Likely Cause, Study Says*, USA Today (Sept. 18, 2019), https://www.usatoday.com/story/news/nation/2019/09/18/number-of-abortions-us-drops-guttmacher-institute-study/2362316001/.

20 See *U.S. Abortion Rate Continues to Decline, Hits Historic Low,* Guttmacher Inst. (Jan. 17, 2017), https://www.guttmacher.org/news-release/2017/us-abortion-rate-continues-decline-hits-historic-low.

21
*Id*.

22 See Guttmacher Inst., *supra* note 15. Although poor Americans have higher rates of unintended pregnancy for a range of reasons, central among them is that they struggle to access contraception. See Michele Troutman, Saima Rafique & Torie Comeaux Plowden, *Are Higher Unintended Pregnancy Rates Among Minorities a Result of Disparate Access to Contraception?*, Contracept Reprod Med 5, no. 16 (2020), https://doi.org/10.1186/s40834-020-00118-5 (describing the factors underlying the disparate rates of unintended pregnancy by race and class). One finds evidence of this struggle in the data on contraceptive use among sexually active women not seeking pregnancy. While 90% of those covered by private health insurance and 87% of those covered by Medicaid use contraception, that figure drops to 81% for those who have no insurance coverage. Megan L. Kavanaugh & Emma Pliskin, *Use of Contraception Among Reproductive-aged Women in the United States*, *2014 and 2016*, Guttmacher Inst. (July, 2020). On the cost of contraception, see Eliana Kosova, *How Much Do Different Kinds of Birth Control Cost Without Insurance?*, Nat’l Women’s Health Network (Nov. 17, 2017), https://nwhn.org/much-different-kinds-birth-control-cost-without-insurance/ (noting that the most effective methods, long-acting implants and devices, cost upwards of $800, and that oral contraceptives can cost up to $600 per year). Cost is not the only barrier to contraception, ranging from cost to personal preferences. For example, Black women tend to report higher rates of dissatisfaction with existing contraceptive options, putting them at further disadvantage in terms of risk of unwanted pregnancy. Andrea V. Jackson, Deborah Karasek, Christine Dehlendorf, and Diana Greene Foster, *Racial and Ethnic Differences in Women’s Preferences for Features of Contraceptive Methods*, 93 Contraception 406–411 (2016).

23 See Colleen L. MacCallum-Bridges & Claire Margerison, *The Affordable Care Act Contraception Mandate & Unintended Pregnancy in Women of Reproductive Age: An Analysis of the National Survey of Family Growth, 2008–2010 v. 2013–2015*, 101 Contraception 34–39 (2020) (Overall, the odds of experiencing unintended pregnancy decreased 15% from the pre-mandate to post-mandate period); See also Susan Christiansen, *The Impact of the Affordable Care Act Contraceptive Mandate on Fertility and Abortion Rates* (Dec. 2020) (Ph.D. dissertation, Johns Hopkins University), https://jscholarship.library.jhu.edu/handle/1774.2/63939 (last visited Jan. 28, 2022).

24 See Molly Jong-Fast, *The Anti-Birth Control Movement Is the New Anti-Abortion Movement*, Vogue (July 1, 2021), https://www.vogue.com/article/anti-birth-control-movement.

25 Burwell v. Hobby Lobby Stores, Inc., 573 U.S. 682 (2014) (striking down the ACA’s contraception mandate because it ‘created a substantial burden’ on Hobby Lobby’s religious freedom and it was not the ‘least restrictive means of satisfying the government’s interests.’)**.** See eg Tom Cohen, *Hobby Lobby Ruling Much More Than Abortion*, Cnn Politics (July 2, 2014), https://www.cnn.com/2014/07/02/politics/scotus-hobby-lobby-impacts/index.html (describing anti-abortion advocates opposition to contraception mandates).

26 See Carole Joffe, *Failing to Embed Abortion Care in Mainstream Medicine Made it Politically Vulnerable*, Wash. Post (Jan. 11, 2022), https://www.washingtonpost.com/outlook/2022/01/11/failing-embed-abortion-care-mainstream-medicine-made-it-politically-vulnerable/ (describing the range in quality of abortion providers when abortion was illegal).

27 See Elizabeth G. Raymond et al., *Efficacy of Misoprostol Alone for First-Trimester Medical Abortion: A Systematic Review*, 133 Obstet Gynecol 133–147 (2019).

28
*Id.* (This consolidated report of existing research finds that misoprostol alone successfully terminates a pregnancy in 93% of cases).

29 See *How to Buy Abortion Pills That Are Safe and Effective*, https://www.ipas.org/our-work/abortion-self-care/abortion-with-pills/how-to-buy-abortion-pills-that-are-safe-and-effective/ (Noting that manufacturers sell the pills to pharmacies for very little cost—less than $0.05 per pill—and that the highest sales price found in a recent study was $2 per pill).

30 See eg Celina Schocken, *Business Case: Investing in Production of High Quality Misoprostol for Low-Resource Settings*, Concept. Found. (Dec. 2014), https://www.conceptfoundation.org/wp-content/uploads/2015/06/BusinessCase_Misoprostol_web.pdf (describing misoprostol’s vital role in treating postpartum hemorrhage); see also *Essential Medicines List includes Misoprostol tablets for use during pregnancy, childbirth and postpartum care*, World Health Organization, https://www.who.int/data/gho/indicator-metadata-registry/imr-details/essential-medicines-list-includes-misoprostol-tablets-for-use-during-pregnancy-childbirth-and-postpartum-care.

31 Beverly Winikoff & Wendy Sheldon, *Use of Medicines Changing the Face of Abortion*, Guttmacher Inst. (Sept. 2012), https://www.guttmacher.org/journals/ipsrh/2012/09/use-medicines-changing-face-abortion. For a description of the misoprostol market in countries where abortion is illegal, see Michelle Oberman, *What Happens When Abortion is Banned?*, N.Y. Times (May 31, 2018), https://www.nytimes.com/2018/05/31/opinion/sunday/abortion-banned-latin-america.html.

32 See Gilda Sedgh et al., *Induced Abortion: Incidence and Trends Worldwide from 1995 to 2008*, 379 The Lancet 625, 625 (2012) (comparing the one out of three pregnancies in Central America end in abortion with one out of five in the United States); see also Susheela Singh et al., *Abortion Worldwide: A Decade of Uneven Progress*, Guttmacher Inst. (Oct. 2009), http://www.guttmacher.org/pubs/Abortion-Worldwide.pdf.

33 There is evidence of an expanding market in misoprostol in the U.S. See Caroline Kitchener, *Self-Managed Abortion Could be the Future—But it’s Very Hard to Talk About*, The Lily (Dec. 20, 2021), https://www.thelily.com/self-managed-abortion-could-be-the-future-but-its-very-hard-to-talk-about/. See also, Erica Hellerstein, *The Rise of the DIY Abortion in Texas*, The Atlantic (June 27, 2014), https://www.theatlantic.com/health/archive/2014/06/the-rise-of-the-diy-abortion-in-texas/373240/.

34 Kari White et al., *Initial Impacts of Texas’ Senate Bill 8 on Abortions in Texas and at Out-of State Facilities*, Texas Policy Evaluation Project (Oct. 2021), http://sites.utexas.edu/txpep/files/2021/11/TxPEP-brief-SB8-inital-impact.pdf.

35 In the immediate aftermath of Prohibition–evidenced by mortality rates, mental health, and crime statistics–alcohol consumption fell to approximately 30% of its pre-Prohibition level. But this drop in alcohol consumption was short-lived. Within a few years after Prohibition, alcohol consumption had increased to 60–70% of its pre-Prohibition level. See Annika Nekalson, *Prohibition was a Failed Experiment in Moral Governance,* The Atlantic (Jan. 16, 2020), https://www.theatlantic.com/ideas/archive/2020/01/prohibition-was-failed-experiment-moral-governance/604972/.

36 See Nash & Dreweke, *supra* note 19, regarding the various reasons behind declining abortion rates.

37 See Greene Foster, *Stop Saying*, *supra* note 6; see also Greene Foster, The Turnaway Study, *supra* note 2.

38 Hence the insistence of the reproductive justice movement that advocates center the goals of racial justice. RJ Squared. For a detailed description of the ways in which structural inequities circumscribe the reproductive choices of poor women and women of color, see Jamila K. Taylor, *Structural Racism and Maternal Health Among Black Women*, 48 Journal Of Law, Medicine & Ethics 506–517 (2020).

39 See generally, Katrina Kimport, No Real Choice: How Culture And Politics Matter For Reproductive Autonomy (2021) (positing that for the most marginalized Americans faced with an unwanted pregnancy, the question is not whether to have an abortion or have a baby, but rather, whether they can actually get an abortion or not).

40 At oral argument, Justice Barrett said, “Both Roe and Casey emphasized the burdens of parenting, and insofar as you and many of your amici focus on the ways in which the forced parenting, forced motherhood would hinder women’s access to the workplace and to equal opportunities, it’s also focused on the consequences of parenting and the obligations of motherhood that flow from pregnancy. Why don’t the safe haven laws take care of that problem?” Transcript of Oral Argument at 56, Dobbs v. Jackson Women’s Health, No. 19–1392. For a compelling analysis and indictment of safe haven laws, see Laury Oaks, Giving Up Baby: Safe Haven Laws, Motherhood, And Reproductive Justice (2015); see also Lizzie Widdicombe, *The Baby-Box Lady of America*, The New Yorker (Dec. 18, 2021) https://www.newyorker.com/news/news-desk/the-baby-box-lady-of-america.

41 See Mardie Caldwell, *Open Adoption is a Win-Win Situation* (Apr. 7, 2018), https://mardiecaldwell.com/open-adoption-is-a-win-win-situation/; see also Julia D. Hejduk, *Gift Motherhood, the Prius, and the Peace Corps: Reducing Abortion by Incentivizing Adoption*, The Public Discourse (Sept. 27, 2017), https://www.thepublicdiscourse.com/2017/09/20054/.

42
*Id*. On the merits, what limited scholarly evidence there is on adoption runs counter to this rosy characterization of adoption’s outcomes, at least for birth mothers. See eg *Are Birth Mothers Satisfied with Decisions to Place Children for Adoption?*, Science Daily (June 8, 2018), https://www.sciencedaily.com/releases/2018/06/180608131605.htm (a longitudinal study of birth mothers that found women reported a mean satisfaction of 3.11 on a scale of 1–5). See also Gretchen Sisson, *‘Choosing Life’: Birth Mothers on Abortion and Reproductive Choice*, 25 Women’s Health Issues 349–54 (2015) (a study involving in-depth interviews with 40 women who had placed infants for adoption from 1962 to 2009. The majority of the participants–many of whom placed their babies in closed adoptions, which are less typical today–described their adoption experiences as ‘predominantly negative,’ a response that Sisson attributes in part to the reality that adoption is not a preferred course of action, but rather, something chosen by those who feel they have no other options).

43 Olga Khazan, *Why So Many Women Choose Abortion Over Adoption*, The Atlantic (May 20, 2019). (https://www.theatlantic.com/health/archive/2019/05/why-more-women-dont-choose-adoption/589759; S*ee also* Olga Khazan, *The New Question Haunting Adoption*, The Atlantic (Oct. 22, 2020), https://www.theatlantic.com/politics/archive/2021/10/adopt-baby-cost-process-hard/620258/ (‘Since the mid-1970s—the end of the so-called baby-scoop era, when large numbers of unmarried women placed their children for adoption—the percentage of never-married women who relinquish their infants has declined from nearly 9% to less than 1%.’).

44 See Elizabeth Wildsmith, Jennifer Manlove et al., *Dramatic Increase in the Proportion of Births Outside of Marriage in the United States from 1990 to 2016*, Child Trends (Aug. 8, 2018), https://www.childtrends.org/publications/dramatic-increase-in-percentage-of-births-outside-marriage-among-whites-hispanics-and-women-with-higher-education-levels#:∼:text=Recent%20estimates%20show%20that%20about, worldwide%20(Chamie%2C%202017). The most rapid growth is among white women: as of 2016, 28% of all births to white women were non-marital. See also Khazan*, supra* note 43 (Starting in the 1970s, single white women became much less likely to relinquish their babies at birth: nearly a fifth of them did so before 1973; by 1988, just 3% did).

45 Khazan, *Id*.

46 The Turnaway Study is a longitudinal study examining the effects of unintended pregnancy on women’s lives. For the study and its findings, see Greene Foster, The Turnaway Study, *supra* note 2.

47 See *id*. See also Diana Greene Foster, *What Happens When It’s Too Late to Get an Abortion*, N.Y. Times (Nov. 22, 2021), https://www.nytimes.com/2021/11/22/opinion/abortion-supreme-court-women-law.html.

48 See Gretchen Sisson, Lauren Ralph, Heather Gould & Diana Greene Foster, *Adoption Decision Making among Women Seeking Abortion, 27* Women’s Health Issues 136 (2017).

49 See *Women’s Access to Abortion Improves Children’s Lives*, Ansirh (Jan. 2019), https://www.ansirh.org/sites/default/files/publications/files/womens_access_to_abortion_improves_childrens_lives.pdf.

50 See generally Kayla Patrick, *National Snapshot: Poverty Among Women & Families*, National Women’s Law Center, https://nwlc.org/wp-content/uploads/2017/09/Poverty-Snapshot-Factsheet-2017.pdf. (The poverty rate for female-headed families with children was 36.5%, compared to 22.1% for male-headed families with children and 7.5% of families headed by married couples with children).

51 Robin Bleiweis, Diana Boesch & Alexandra C. Gaines, *The Basic Facts About Women in Poverty*, American Progress (Aug. 3, 2020), https://www.americanprogress.org/article/basic-facts-women-poverty/.

52 See American Academy of Pediatrics, Council on Community Pediatrics, *Poverty and Child Health in the United States*, Pediatrics 137 no. 4 (2016): e20160339.

53 See kimport, *supra* note 39, at 37, and generally.

54 See Leah Outten, *Birth Mothers and the Adoption Option*, Focus On The Family (Nov. 9, 2021), https://www.focusonthefamily.com/pro-life/the-adoption-option-birth-mothers-need-your-support/; Stephanie McCrummen, *A Maternity Ranch is Born: How Evangelical Women in Texas are Mobilizing for a Future Without Abortion*, Wash. Post (Nov. 16, 2021), https://www.washingtonpost.com/nation/2021/11/16/evangelical-women-texas-abortion/?utm_campaign=wp_post_most&utm_medium=email&utm_source=newsletter&wpisrc=nl_most&carta-url=https%3A%2F%2Fs2.washingtonpost.com%2Fcar-ln-tr%2F354c967%2F6193e9909d2fdab56b8e7f16%2F5a1f90909bbc0f4d5203bb72%2F8%2F73%2F6193e9909d2fdab56b8e7f16. see also Michelle Oberman, *The Women the Abortion War Leaves Out*, N.Y. Times (Jan. 11, 2018), https://www.nytimes.com/2018/01/11/opinion/sunday/abortion-crisis-pregnancy-centers.html (describing Oklahoma City’s Rose Home, a pro-life organization that houses up to five pregnant women and their children at any given time).

55 See Outten, *supra* note 54. See also Eleanor Bartow, *12 Pro-Life Truths to Counter Every Abortion Myth*, The Federalist (Oct. 11, 2021) https://thefederalist.com/2021/10/11/12-pro-life-truths-to-counter-every-abortion-myth/ (where Bartow asserts adoption is ‘a better option than killing an unborn child’ because there are many ‘loving, screened, financially stable parents [who] are waiting to adopt babies.’).

56 See Sue Halpern, *How Republicans Became Anti-Choice*, The N.Y. Rev. (Nov. 8, 2018), https://www.nybooks.com/articles/2018/11/08/how-republicans-became-anti-choice/ (reviewing *Reversing Roe*, a documentary film directed and produced by Ricki Stern and Annie Sundberg).

57 See Robert Reich, *Republicans, So Called party of Family Values, Do Not Support Needy Families*, The Guardian (Jul. 18, 2021), https://www.theguardian.com/commentisfree/2021/jul/18/child-allowance-payments-american-rescue-plan-republicans

58 See eg Richard Garnett, *supra* note 4. See also Hadley Arkes, in *One Untrue Thing*, Nat’l Rev. (Aug. 1, 2007), https://www.nationalreview.com/2007/08/one-untrue-thing-nro-symposium/, (‘[T]he law does not need to invoke the harshest penalties for the sake of teaching moral lessons.’).

59 §315, Penal Law, 5737–1977, LSI Special Volume (1977), as amended (Isr.),
https://knesset.gov.il/review/data/eng/law/kns8_penallaw_eng.pdf [https://perma.cc/N9QZ-8JEV].

60 Or at least it would do so if the law were interpreted in a way that strictly limited abortion access. Instead, as I explain elsewhere, the committees close to 100% of the requests they receive, making legal abortion readily available in the country. See Oberman, *supra* note 2, at 172.

61 And they do. Recall that Israel has the highest fertility rates of any country in the OECD. Families have an average of 3.1 children. See *Family Database: Fertility Rates*, Org. Econ. Coop. Dev., https://www.oecd.org/els/family/SF_2_1_Fertility_rates.pdf [https://perma.cc/5J2Y-YGWG] (last updated June 2021).

62 See *Maternity Leave*, Kol Zchut, https://www.kolzchut.org.il/en/Maternity_Leave [https://perma.cc/Q29Q-T6Q6] (last visited June 19, 2021).

63 See *Childcare in Israel*, Expat.Com (Sept. 18, 2017) https://www.expat.com/en/guide/middle-east/israel/15420-childcare-in-israel.html [https://perma.cc/2Z7S-SJCK] (describing the relative level of state support that young Israeli families receive, compared to the U.S.). See also, *Register to State Recognized Daycare and Afternoon Care, and Request State Participation in Tuition Fees*, Gov. IL, https://www.gov.il/en/service/registration_for_day_care_centers_and_nurseries1 (last updated Feb. 7, 2020) (Israeli government website describing eligibility for state supported day care). Rates of enrollment in both day care and preschool are among the highest in the developed world. Indeed, preschool enrollment rates are double that of the OECD average. https://issuu.com/bernardvanleerfoundation/docs/publication_taub_center_early_childhood_education_ (at 27).

64 See, eg *Children*, Nat’l Ins. Inst. Of Isr., https://www.btl.gov.il/English%20Homepage/Benefits/Children/Pages/default.aspx [https://perma.cc/8KE2-3K8L] (last visited on Dec. 19, 2021). Although these policies may not significantly offset the costs of having a child, surely, they are a benefit to Israel’s poorest families.

65 The only government support for those who have babies lies in family medical leave, which promises twelve weeks of unpaid leave time after the birth of a child. See Family and Medical Leave Act (FMLA), 29 U.S.C. § 2612.

66 Ben Casselman, *Child Tax Credit’s Extra Help Ends, Just as Covid Surges Anew*, N.Y. Times (last updated Jan. 3, 2022), https://www.nytimes.com/2022/01/02/business/economy/child-tax-credit.html.

67
*Id*.

68 Ali Safawi & Cindy Reyes, *States Must Continue Recent Momentum to Further Improve TANF Benefit Levels*, Cntr on Budget & Pol’y Priorities (updated Dec. 2, 2021), https://www.cbpp.org/research/family-income-support/states-must-continue-recent-momentum-to-further-improve-tanf-benefit.

69 For signs that anti-abortion advocates are beginning to grapple with the need to take systemic realities into account, see Tish Harrison Warren, *The Systemic Realities Created by Legal Abortion*, N.Y. Times (Jan. 22, 2022), https://www.nytimes.com/2022/01/22/opinion/roe-legal-abortion.html.

70 See Richard H. McAdams*, A Focal Point Theory of Expressive Law,* 86 V.A. L. Rev.1649, 1713–28 (2000) (applying expressive law theory to smoking bans and landlord liability law). See generally Richard H. McAdams, The Expressive Powers Of Law: Theories And Limits (2015).

71 He offers little evidence, by way of proof. However, numerous studies both domestically and worldwide document an association between smoking bans and an overall decline in smoking rates, including a reduction in smoking by smokers. See eg Thomas W. Carton, Michael Dardon, et al., *Comprehensive Indoor Smoking Bans and Smoking Prevalence: Evidence from the BRFSS*, 2 J Health Econ. 535–56 (2016); see also Silke Anger, Michael Kvasnicka, Thomas Seidler, *One Last Puff? Public Smoking Bans and Smoking Behavior*, 30 J Health Econ. 591–601 (2011).

72 See McAdams, *supra* note 70, at 197.

73 These lawmakers also must contend with considerable public opposition to their position. As of 2020, 79% of Americans say that the decision to have an abortion is best left to women, not lawmakers, according to a Kaiser Family Foundation study from 2020. See Ariana Eunjung Cha & Emily Guskin, *Most Americans Want Abortion to Remain Legal, but Back S ome State Restrictions*, Wash. Post (Jan. 22, 2020), https://www.washingtonpost.com/health/2020/01/22/most-americans-want-abortion-remain-legal-back-some-state-restrictions/.

74 See McAdams, *supra* note 70, at 180.

75 Recreational cannabis is legal in 18 states, while 11 states criminalize it. (See https://disa.com/map-of-marijuana-legality-by-state for a breakdown of the various jurisdictions’ laws). Experts estimate that at least 15 states will keep abortion legal, and perhaps even expand abortion rights, regardless of the absence of a Constitutional right. https://www.guttmacher.org/state-policy/explore/abortion-policy-absence-roe.

76 Kimport, *supra* note 37, at 28 and 62–69.

77 See eg Anna North, *All the Near-Total Abortion Bans Passed This Year Have Now Been Blocked in Court,* Vox (updated Oct. 29, 2019), https://www.vox.com/2019/10/2/20895034/alabama-abortion-ban-blocked-georgia-law; see also Sean Murphy, *Oklahoma Supreme Court Blocks 3 New Anti-Abortion Laws,* ABC News (Oct. 25, 2021), https://abcnews.go.com/Health/wireStory/oklahoma-supreme-court-blocks-anti-abortion-laws-80779946.

78 See Chotiner, *supra* note 5. See also O. Carter Snead, in *One Untrue Thing*, Nat’l Rev. (Aug. 1, 2007), https://www.nationalreview.com/2007/08/one-untrue-thing-nro-symposium/ (Offering a pragmatic justification for not punishing self-abortion: ‘[T]he public is more willing to accept a law that punishes doctors rather than mothers. Pro-lifers can thus achieve their goal of ending abortion without provoking a political backlash.’).

79 Although there are legal strategies a government might employ in response to overseas entities that sell abortion medicines to U.S. consumers (eg border patrol agents or diplomatic pressure), we learn from both the heroin and the fentanyl epidemics that the government’s options in the face of high demand are limited. See Claire Felter, *Backgrounder: The U.S. Opioid Epidemic*, The Council on Foreign Relations, (Sept. 8, 2021), https://www.cfr.org/backgrounder/us-opioid-epidemic.

80 Stephanos Bibas, *Prosecutorial Regulation Versus Prosecutorial Accountability*, 157 U. Pa. L. Rev. 959 (2009).

81 Stephanos Bibas, *The Need for Prosecutorial Discretion*, 19 Temp. Pol. & Civ. Rts. Rev. 369, 371 (2010).

82 See Leslie Reagan, When Abortion Was A Crime: Women, Medicine, And Law In The United States, 1867–1973 (1997), at 114, 164.

83 See *From Hospital to Jail: The Impact on Women of El Salvador’s Total Criminalization of Abortion*, 22 Repr. Health Matters 52–60 (2014); see also Oberman, *supra* note 2*,* at 8–10 and 49–55 (describing similar patterns in Chile and El Salvador).

84 See Oberman*, supra* note 2, at 44.

85
*Id*.

86 See Repr. Health Matters, *supra* note 83.

87 See eg Chelsea Becker’s prosecution for murder, following stillbirth allegedly caused by methamphetamine use. *Judge Dismisses Murder Charge Against Califronia Mother After Stillbirth*, N.Y. Times (May 21, 2021), https://www.nytimes.com/2021/05/20/us/chelsea-becker-stillbirth-murder-charges-california.html.

88 They may also conscript doctors into law enforcement. See Michelle Oberman, *Abortion Bans, Doctors, and the Criminalization of Patients*,48 Hastings Ctr. Rep.5 (2018); see also Oberman, Her Body Our Laws, *supra* note 2, at 43–67 for a discussion of how reports from doctors to police in El Salvador overwhelmingly involve poor, marginalized women.

89 Priscilla Thompson & Alexandra Turcios Cruz, *How an Oklahoma Women’s Miscarriage Put a Spotlight on Racial Disparities in Prosecutions*, Nbc News (Nov. 5, 2021), https://www.nbcnews.com/news/us-news/woman-prosecuted-miscarriage-highlights-racial-disparity-similar-cases-rcna4583. For a detailed discussion of these cases, see Lynn M. Paltrow & Jeanne Flavin, *Arrests of and Forced Interventions on Pregnant Women in the United States, 1973–2005: Implications for Women’s Legal Status and Public Health*, 38 J. Health Pol., Pol’y And L. 299, 304–05 (2013) (discussing these findings and the limitations of the research which led the authors to conclude that their findings represent a substantial undercount of cases). See also Michele Goodwin, Policing The Womb: Invisible Women And The Criminalization Of Motherhood (2020).

90 See *Arrests and Prosecutions of Pregnant Women, 1973–2020*, Napw (Sept.18, 2021), https://www.nationaladvocatesforpregnantwomen.org/arrests-and-prosecutions-of-pregnant-women-1973-2020/(summarizing the range of cases). See also Lynn M. Paltrow, *Constitutional Rights for the ‘Unborn’ Would Force Women to Forfeit Theirs*, Ms. Magazine (Apr. 15, 2021), https://msmagazine.com/2021/04/15/abortion-constitutional-rights-unborn-fetus-14th-amendment-womens-rights-pregnant/ (The rate of arrests and prosecutions is increasing. ‘From 2006–2020, we have documented over 1000 such arrests—more than double in half as many years. Black, Brown and low-income, rural white women are the typical targets of these arrests.’).

91 Thompson & Turcios Cruz, *supra* note 85 (noting that the Black defendants were also significantly more likely to be charged with felonies than white women, with 85% of Black women receiving felony charges compared to 71% of white women); see also Lynn M. Paltrow, *Roe v. Wade and the New Jane Crow: Reproductive Rights in the Age of Mass Incarceration*, 103 Am. J. Pub. Health 17, 19 (2013). Note that healthcare experts object strenuously to these prosecutions on the grounds that they deter people from seeking treatment essential both to their own welfare and to that of the fetus. See eg Katherine C. Arnold, *Viewpoint: Criminalizing Young Women is not the Way to Improve Birth Outcomes*, The Oklahoman (Dec. 26, 2021, 5:00 AM), https://www.oklahoman.com/story/opinion/2021/12/26/viewpoint-prosecuting-oklahoma-women-who-miscarry-wrong/8930865002/.

92 While beyond the scope of this Article, it bears noting the range of options that state lawmakers have given prosecutors, when it comes to abortion crimes, outlawing things like purchasing abortion medicine, or aiding and assisting an abortion. See eg Emily Bazelon, *A Mother in Jail for Helping her Daughter Have an Abortion*, N.Y. Times (Sept. 22, 2014), https://www.nytimes.com/2014/09/22/magazine/a-mother-in-jail-for-helping-her-daughter-have-an-abortion.html. See also Sabrina Tavernese, *Citizens, not the State, Will Enforce New Abortion Law in Texas*, N.Y. Times (Nov. 1, 2021), https://www.nytimes.com/2021/07/09/us/abortion-law-regulations-texas.html (Describing the ways that Texas S.B. Eight criminalizes all those who aid and assist abortion, and quoting Prof. Melissa Murray, ‘If the barista at Starbucks overhears you talking about your abortion, and it was performed after six weeks, that barista is authorized to sue the clinic where you obtained the abortion and to sue any other person who helped you, like the Uber driver who took you there.’).

93 See Paltrow*, supra* note 91. See generally Michele Bratcher Goodwin, *Invisible Women: Mass Incarceration’s Forgotten Casualties*, 94 Tex. L. Rev. (2015).

94 See *Race & Justice News: Eliminating Crack/Cocaine Sentencing Disparity* The Sentencing Project (July 27, 2021), https://www.sentencingproject.org/news/race-justice-news-senate-hearing-crack-cocaine-sentencing-disparity/ (summarizing the ongoing work toward sentencing equality in drug crimes, starting with the 2010 Fair Sentencing Act). On the negative downstream consequences of race bias in drug law enforcement, Republican Governor Asa Hutchinson noted that racial disparities, “undermined community confidence in the fairness of the criminal justice system. I talked with drug task force officers and front-line agents at the DEA who said this sense of injustice had a real impact in the fight against illegal drugs; it made it more difficult for agents to build trust and work with informants in the areas most impacted by the crack epidemic. The disparity in sentencing led to more harm than help in our federal anti-crime efforts.” (*Gov. Asa Hutchinson: It’s Time to Fix an Old Wrong and End the Disparity Between Crack and Cocaine Offenses*, Fox News (June 8, 2021)), https://www.foxnews.com/opinion/end-crack-cocaine-offenses-gov-asa-hutchinson.

95 See Caroline Kitchener, *The Texas Abortion ban has a Medical Exception. But some Doctors Worry it’s too Narrow to use,* The Lily, Oct. 22, 2021 (describing existing legal protections and the limitations of Texas S.B. 8’s ‘medical emergency’ exception to its abortion ban), https://www.thelily.com/the-texas-abortion-ban-has-a-medical-exception-but-some-doctors-worry-its-too-narrow-to-use/.

96 See Michael Asimow, Federal, Administrative Adjudication Outside The Administrative Procedure Act 3–4 (2019) (classifying these hearings into three categories, according to level of formal process).

97 See Kari White, Subasri Narasimhan, Sophie A. Hartwig, Erin Carroll, Alexandra McBrayer, Samantha Hubbard, Rachel Rebouché, Melissa Kottke & Kelli Stidham Hall, *Parental Involvement Policies for Minors Seeking Abortion in the Southeast and Quality of Care*, Sexuality Rsch. & Soc. Pol’y (Jan. 18, 2021), https://link.springer.com/content/pdf/10.1007/s13178-021-00539-0.pdf.

98 See David S. Cohen & Carole Joffe, Obstacle Course: The Everyday Struggle To Get An Abortion In America 209 (2020) (quoting Ohio doctor Chrisse France, decrying this standard in U.S. practice today; ‘She cannot be seen at our public hospital unless pretty much she’s going to die today or maybe tomorrow’).

99 See Kari White, Subasri Narasimhan, Sophie A. Hartwig, Erin Carroll, Alexandra McBrayer, Samantha Hubbard, Rachel Rebouché, Melissa Kottke & Kelli Stidham Hall, *Parental Involvement Policies for Minors Seeking Abortion in the Southeast and Quality of Care*, Sexuality Rsch. & Soc. Pol’y (Feb., 2021) (noting the impact of these policies in delaying access to early abortion among those ultimately deemed eligible to end their pregnancies).

100 See Herbert L. Packer & Ralph J. Gampell, *Therapeutic Abortion: A Problem in Law and Medicine*, 11 Stan. L. Rev. 417, 418, 421 (1959). (Explaining that hospitals developed protocols at least in part as a defensive measure: to protect themselves from potential downstream criminal or civil liability). See also, Carole Joffe, Doctors Of Conscience 31 (1995) (describing how doctors who performed abortions illegally would do so outside of the hospital setting, but legal abortions that met the test of necessary to save life would necessarily have been performed in a hospital, thereby implicating both medical and hospital oversight).

101 See generally Packer & Gampell, *id*. at *418*. (Noting these questions, among others: Is the procedure limited to cases where its purpose is to avoid shortening the pregnant woman’s life? If so, how do we determine whether carrying the child to term will shorten life? If not, what other considerations are relevant? Is a threat to health necessarily a threat to life? Must the threat to life (or health) be on account of a somatic illness? Or is the woman’s mental condition also to be considered? If so, is a probability that suicide will ensue a justification for therapeutic abortion?). For a searing indictment of U.S. therapeutic abortion committee practices in the mid-twentieth century, see Rickie Solinger, *‘A Complete Disaster:’ Abortion and the Politics of Hospital Abortion Committees, 1950–1970*, 19 Feminist Stud. 241 (1993).

102 See Packer & Gampell, *supra* note 100, at 449. (‘[R]eputable members of the medical profession may well find it galling that their freedom from criminal and civil liability turns merely on the nonenforcement of provisions of the law which, on their face, appear to embrace the conduct in question.’). Texas’s S.B. 8 law employs such a threat by way of subjecting doctors who provide abortions after six weeks to civil suit. See Sabrina Tavernese, *supra* note 75.

103
*Id.* at 421 (citing Alan F. Guttmacher, *The Shrinking Non-Psychiatric Indications for Therapeutic Abortion*, in Therapeutic Abortion 12 (Rosen, ed. 1954)).

104
*Id*. at 430. Their study concluded with a call for law reform—a call that was echoed by their Canadian counterparts in the 1977 Badgley report, which found “gross inequities existed in the availability of therapeutic abortion to the women of Canada.” W.D.S. Thomas, The Badgley Report on the Abortion Law, 116 Can. Med. Ass’n. J. 966, 966 (1977).

105 See Carole Joffe and Jody Steinauer, *Evan Texas Allows Abortions to Protect a Woman’s Life. Or Does It?*, N.Y. Times (Sept. 12, 2021), https://www.nytimes.com/2021/09/12/opinion/abortion-texas-roe.html (describing how contemporary abortion bans will likewise challenge medical integrity).

106 See Oberman, *supra* note 2, at 13–42 (describing the highly public Salvadoran case of Beatriz, a woman forced to continue a life-threatening pregnancy until her doctors agreed that death was imminent, which then triggered her right to self-defense, permitting doctors to end her pregnancy).

107 For a thoughtful consideration of the constitutional protections owed to one who is pregnant, when abortion is illegal, see Meghan Boone, *Reproductive Due Process*, 88 Geo. Wash. L. Rev. 511, 526 (2020) (‘Beyond its flexibility and ability to evolve, a third feature of due process is simply its function as a catchall constitutional backstop for determining the fairness of government action’).

108 See Matthews v. Eldridge, 424 U.S. 319, 335 (1976) (“Identification of the specific dictates of due process generally requires consideration of three distinct factors: first, the private interest that will be affected by the official action; second, the risk of erroneous deprivation of such interest through the procedures used, and probable value, if any, of additional or substitute procedural safeguards; and, finally, the Government’s interest, including the function involved and the fiscal and administrative burdens that the additional or substitute procedural requirements would entail.”). See also Simona Grossi, *Procedural Due Process*, 13 Seton Hall Cir. Rev. 155, 158 (2017) (‘[A]…procedural law that is not supported by logic, fairness, and efficiency considerations,…violates due process.’).

109 See Chotiner, *supra* note 5, (citing Marjorie Dannenfelser).

110 See eg Box v. Planned Parenthood, 139 S. Ct. 1780 at 1783 (Thomas, J., concurring) (supporting an Indiana law banning abortion on grounds of fetal anomaly by invoking the state’s ‘compelling interest in preventing abortion from becoming a tool of modern-day eugenics.’).

111 See eg Eli Rosenberg, *Clarence Thomas Tried to Link Abortion to Eugenics. Seven Historians Told the Post He’s Wrong*, Wash. Post (May 30, 2019, 9:50 PM), https://www.washingtonpost.com/history/2019/05/31/clarence-thomas-tried-link-abortion-eugenics-sevenhistorians-told-post-hes-wrong [https://perma.cc/5DNR-PJT5]; Imani Gandy, *When It Comes to Birth Control and Eugenics, Clarence Thomas Gets It All Wrong*, Rewire (May 29, 2019, 5:11 PM), https://rewire.news/ablc/2019/05/29/when-it-comes-to-birth-control-and-eugenics-clarencethomas-gets-it-all-wrong [https://perma.cc/3HZ3-689B]; Lydia O’Connor, *What Justice Clarence Thomas Gets Wrong About Eugenics and Abortion*, Huff. Post (May 29, 2019, 5:50 PM), https://www.huffpost.com/entry/clarence-thomas-eugenics-abortion_n_5ced6c87e4b0356205a07182 [https://perma.cc/6AHJ-MS5U].

112 See Melissa Murray, *Race-ing Roe: Reproductive Justice, Racial Justice, and the Battle for Roe v. Wade*, 134 Harv. L. Rev. 2025 (2021) (providing a compelling response to the eugenics charge); see also Jennifer L. Holland, Tiny You: A Western History Of The Anti-Abortion Movement (2020) (arguing that the goals of the anti-abortion movement are deeply entwined with those of the white supremacy movement). Indeed, one might find evidence of racism in the suggestion that adoption is the best solution for poor Black and brown babies, which echoes the U.S. Indian Adoption Project of 1958–1967, under which as many as one-third of indigenous children were separated from their families. 85% of those children were placed in non-native homes or institutions. See Stephanie Woodward, *Native Americans Expose The Adoption Era and Repair Its Devastation*, Indian Country Today (updated Sept. 13, 2018), https://indiancountrytoday.com/archive/native-americans-expose-the-adoption-era-and-repair-its-devastation.

113
*Reproductive Justice–Sister Song*  https://www.sistersong.net/reproductive-justice. See also, *Mission and Vision*, If, When, How, https://www.ifwhenhow.org/about/mission-vision/ [https://perma.cc/3RRJ-LKT5] (last visited Dec. 21, 2021) (A leading reproductive justice organization, their vision statement reads: ‘We envision a transformation of the legal systems and institutions that perpetuate oppression into structures that realize justice, and a future when all people can self-determine their reproductive lives free from discrimination, coercion, or violence.’).

114 They might begin by looking to Germany. Not, as Chief Justice Roberts suggested in the *Dobbs* oral argument, because its law restricting abortion to 12 weeks’ gestation is a good compromise. (Transcript of Oral Argument at 53–55, Dobbs v. Jackson Women’s Health, No. 19-1392). Rather, because of the story of how anti-abortion German lawmakers concluded that the best way to deter abortion was to enact, along with a partial ban, “a suite of services that had to be made available to women and families as part of any law regulating abortion: financial assistance for stay-at-home parents; a guaranteed return to a parent’s prior job if he or she took off up to three years to care for a child; extended day care and extensive tax credits for day care costs; increases for child support payments; extended paid leave to care for sick children; reemployment guarantees for empty nesters; sex education services; and a host of other measures relating to adoption, housing and taxation.” Jamal Greene, How Rights Went Wrong: Why Our Obsession With Rights Is Tearing America Apart 130 (2021).

115 See Chotiner interview, *supra* note 5. (“We make sure there’s child care in the first few years of that child’s life that’s cheap or free. That’s going to look very different in Minnesota than it does in Georgia. In Minnesota, there very well may be a political appetite for passing more state-supported aid…. There is not a one-size-fits-all when it comes to…how the needs of women will be met. That is vital work for the pro-life movement, and the Republican Party.”).

116 See Isaac Chotiner, *How Pandemics Change History*, The New Yorker (Mar. 3, 2020), https://www.newyorker.com/news/q-and-a/how-pandemics-change-history (quoting Frank M. Snowden).

